# Identification and properties of proteases from an *Acanthamoeba *isolate capable of producing granulomatous encephalitis

**DOI:** 10.1186/1471-2180-6-42

**Published:** 2006-05-03

**Authors:** James Sissons, Selwa Alsam, Graham Goldsworthy, Mary Lightfoot, Edward L Jarroll, Naveed Ahmed Khan

**Affiliations:** 1 School of Biological and Chemical Sciences, Birkbeck College, University of London, England, UK; 2 Department of Biology, Northeastern University, Boston, MA, USA

## Abstract

**Background:**

Granulomatous amoebic encephalitis due to *Acanthamoeba *is often a fatal human disease. However, the pathogenesis and pathophysiology of *Acanthamoeba *encephalitis remain unclear. In this study, the role of extracellular *Acanthamoeba *proteases in central nervous system pathogenesis and pathophysiology was examined.

**Results:**

Using an encephalitis isolate belonging to T1 genotype, we observed two major proteases with approximate molecular weights of 150 KD and 130 KD on SDS-PAGE gels using gelatin as substrate. The 130 KD protease was inhibited with phenylmethylsulfonyl fluoride (PMSF) suggesting that it is a serine protease, while the 150 KD protease was inhibited with 1, 10-phenanthroline suggesting that it is a metalloprotease. Both proteases exhibited maximal activity at neutral pH and over a range of temperatures, indicating their physiological relevance. These proteases degrade extracellular matrix (ECM), which provide structural and functional support to the brain tissue, as shown by the degradation of collagen I and III (major components of collagenous ECM), elastin (elastic fibrils of ECM), plasminogen (involved in proteolytic degradation of ECM), as well as casein and haemoglobin. The proteases were purified partially using ion-exchange chromatography and their effects were tested in an *in vitro *model of the blood-brain barrier using human brain microvascular endothelial cells (HBMEC). Neither the serine nor the metalloprotease exhibited HBMEC cytotoxicity. However, the serine protease exhibited HBMEC monolayer disruptions (trypsin-like) suggesting a role in blood-brain barrier perturbations.

**Conclusion:**

Overall, these data suggest that *Acanthamoeba *proteases digest ECM, which may play crucial role(s) in invasion of the brain tissue by amoebae.

## Background

*Acanthamoeba *are opportunistic protozoans that are widely distributed in the environment. Given the opportunity and the host immune status, pathogenic *Acanthamoeba *can invade the human central nervous system (CNS) and produce fatal granulomatous encephalitis [1-4]. *Acanthamoeba *granulomatous encephalitis (AGE) is characterized by headache, fever, behavioural changes, hemiparesis, lethargy, stiff neck, aphasia, ataxia, vomiting, nausea, cranial nerve palsies, increased intracranial pressure, seizures and ultimately death. Death is due to haemorrhaging necrotic lesions with severe meningeal irritation and encephalitis. The lesions due to AGE are most numerous in the basal ganglia, midbrain, brainstem, and cerebral hemispheres with characteristic lesions in the CNS parenchyma resulting in chronic granulomatous encephalitis.

The mechanisms associated with the pathogenesis of AGE remain incompletely understood, however the pathophysiological complications involving the CNS most likely include induction of the pro-inflammatory responses, invasion of the blood-brain barrier and the connective tissue and neuronal damage leading to the brain dysfunction [1-4]. The routes of entry include lower respiratory tract leading to amoebae invasion of the intravascular space, followed by the haematogenous spread. Skin lesions may provide direct amoebae entry into the bloodstream, thus bypassing the lower respiratory tract. Amoebae entry into the CNS most likely occurs at the sites of the blood-brain barrier [1,2]. In addition, olfactory neuroepithelium provides another route of entry into the CNS and has been studied in experimental models [1,2]. Following CNS invasion, amoebae penetrate the brain tissue to produce disease. Previous studies have shown that proteases play important roles in microbial pathogenesis including host cell and tissue invasion, migration, catabolism of host proteins, cytoadherence, and both stimulation and evasion of host responses [reviewed in [7]]. The present study examines *Acanthamoeba *proteases released extracellularly by an AGE isolate to begin to ask if these proteases could be involved in this amoeba's ability to invade the CNS. Our studies are feasible with the availability of the *in vitro *cultures of the human brain microvascular endothelial cells, which constitute the blood-brain barrier. Here, we report that proteases from an AGE isolate of *Acanthamoeba *digest ECM and produce blood-brain barrier disruptions and thus may play crucial role(s) in invasion of the brain tissue by amoebae.

## Results

### *Acanthamoeba *keratitis isolate (T4 genotype) exhibited two serine proteases while AGE isolate (T1 genotype) exhibited one serine and one metalloprotease

**Figure 1:**
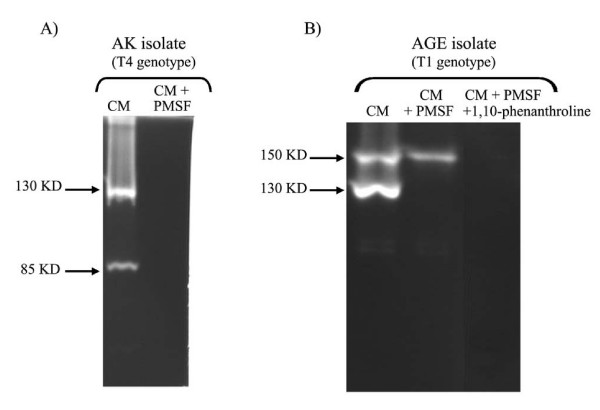
***Acanthamoeba *extracellular protease profiles of T1 (AGE isolate) and T4 (AK isolate)**. Conditioned medium (CM) from T1 and T4 isolates was collected and used for zymography as described in "Materials and Methods". CM + PMSF or CM + 1, 10-phenathroline means that the CM was incubated with these protease inhibitors prior to electrophoresis and in the case of 1, 10 phenanthroline it was also included in the developing buffer. Note that AK isolate (T4 genotype) exhibited two serine proteases of approx. Mw. of 85 KD and 130 KD that were inhibited with PMSF, i.e., serine protease inhibitor. In contrast, AGE isolate exhibited one serine protease at approx. Mw. 130 KD and a 150 KD metalloprotease sensitive to 1, 10-phenanthroline, i.e., metalloprotease inhibitor. Results are representative of three independent experiments.

To determine the extracellular proteases of *Acanthamoeba*, conditioned medium (CM) was used for zymographic assays. *Acanthamoeba *keratitis isolate (T4 genotype) exhibited two protease bands observed at approximate molecular weights (Mw) of 130 KD and 85 KD (Fig. 1A). However, both protease bands were sensitive to PMSF indicating that *Acanthamoeba *secrete serine proteases, a finding that is consistent with previous studies (reviewed in [5]). Thus, the remaining studies have been focused on the AGE isolate. In contrast, AGE isolate (T1 genotype) exhibited a 150 KD metalloprotease (inhibited with 1, 10-phenanthroline) and a 130 KD serine protease (inhibited by PMSF) (Fig. 1B). Of interest, CM produced from co-cultures of *Acanthamoeba *and HBMEC exhibited similar protease profiles (data not shown).

### Conditioned medium of the AGE isolate exhibited HBMEC monolayer disruptions but not cytotoxicity

**Figure 2:**
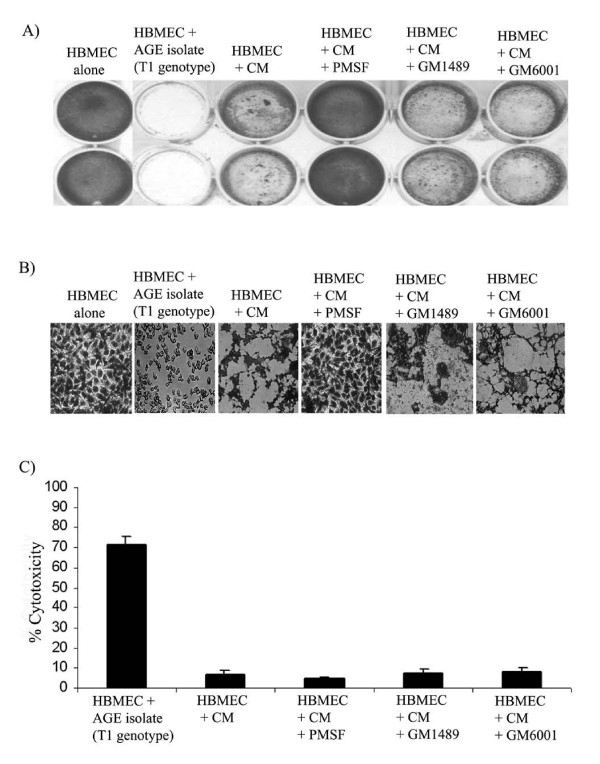
**A) Conditioned medium (CM) of the AGE isolate exhibited HBMEC monolayer disruptions**. *Acanthamoeba *(AGE isolate belonging to T1 genotype) or their CM were added to human brain microvascular endothelial cells (HBMEC) monolayers as described in "Materials and Methods". **A) **HBMEC monolayers were stained with hematoxylin to observe monolayer disruptions. HBMEC alone represent HBMEC without parasites and/or CM and were used as controls. Both *Acanthamoeba *as well as CM exhibited HBMEC monolayer disruptions. Note that PMSF blocked CM-mediated HBMEC monolayer disruptions. In contrast, metalloprotease inhibitors (GM 1489 and GM 6001) had no effects on CM-mediated HBMEC monolayer disruptions. **B) **Micrography of HBMEC cytopathic effects. Micrographs were taken using a phase-contrast microscope, ×200. **C) **CM of the AGE isolate did not produce HBMEC cytotoxicity. Note that *Acanthamoeba *(AGE isolate belonging to T1 genotype) exhibited more than 70% HBMEC cytotoxicity, while their CM induced less than 10% HBMEC cytotoxicity. Results are mean of three experiments performed in duplicates. Error bars represent standard error.

To determine the cytopathic effects of extracellular proteases of AGE isolate (T1 genotype), cytotoxicity assays were performed. Hematoxylin staining of HBMEC monolayers revealed that the AGE isolate as well as its CM produced total HBMEC monolayer disruptions (Fig. 2A and 2B). The CM-mediated HBMEC monolayer disruptions were completely abolished after pre-incubating the CM with PMSF (Fig. 2A and 2B). Pre-incubations with broad spectrum matrix metalloprotease (MMP) inhibitors, i.e., GM 1489 or GM 6001, had no effect on CM-mediated HBMEC monolayer disruptions (Fig. 2A and 2B). The AGE isolate produced more than 70% HBMEC cytotoxicity, while its CM exhibited minimal HBMEC cytotoxicity (Fig. 2C). Furthermore, neither broad spectrum MMP inhibitors had an effect on AGE isolate-mediated HBMEC cytotoxicity (data not shown). Similar findings were observed with AK isolate belonging to T4 genotype (data not shown). Taken together, these data demonstrate that 130 KD serine protease (but not the 150 KD metalloprotease) produced HBMEC monolayer disruptions but not HBMEC cytotoxicity.

### The effect of various temperatures and pH on the extracellular proteases of AGE isolate

**Figure 3:**
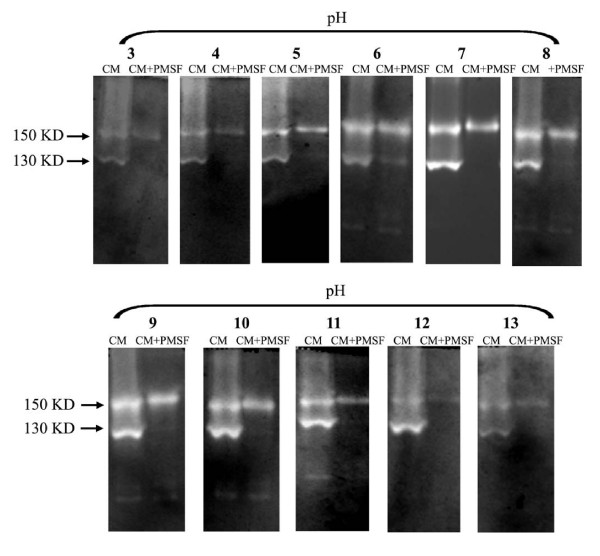
***Acanthamoeba *(AGE isolate belonging to T1 genotype) proteases exhibit optimal activities at neutral pH**. To determine the effects of pH, zymographic assays were performed as described in the "Materials and Methods" and gels incubated at various pHs. Note that neutral pH produced optimal proteolytic activities. Results are representative of three independent experiments. Each lane contained 0.5 μg protein.

**Figure 4:**
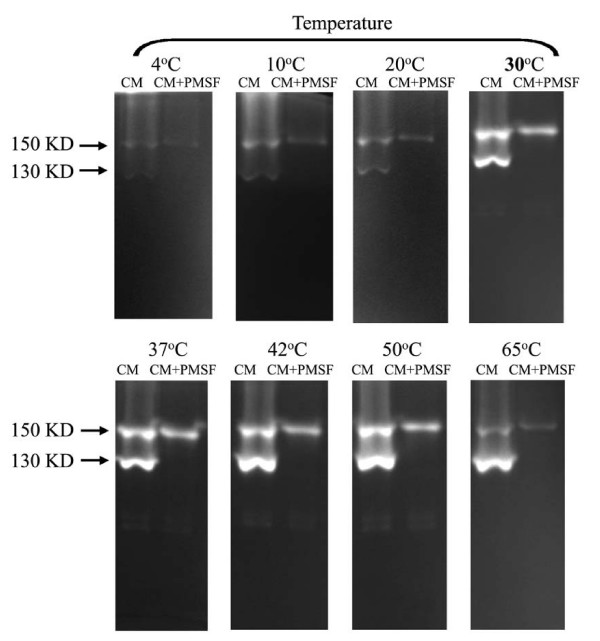
***Acanthamoeba *(AGE isolate belonging to T1 genotype) proteases exhibit optimal activities at various temperatures**. To determine the effects of variable temperatures, zymographic assays were performed as described in the "Materials and Methods" and gels incubated at various temperatures. Note that 37 – 50°C produced optimal proteolytic activities. Results are representative of three independent experiments. Each lane contained 0.5 μg protein.

To determine the physiological properties of extracellular proteases of AGE isolate (T1 genotype), zymographic assays were performed using gelatin as substrate and gels incubated at various temperatures and pH. The results revealed that both serine and metalloproteases exhibited optimal activities at neutral pH (Fig. 3). The influence of various temperatures on the proteolytic activities was tested in the temperature intervals from 4°C – 65°C. The optimal activity was observed at temperatures varying from 37°C – 50°C (Fig. 4), indicating that proteases are also active at physiologically relevant conditions. Of interest, the metalloprotease (approx. Mw. 150 KD) visually appeared to have lost significant activity in zymography during incubation at 65°C (Fig. 4).

### Both serine (130 KD) and metalloproteases (150 KD) of AGE isolate exhibited collagenase and elastinolytic activities

**Figure 5:**
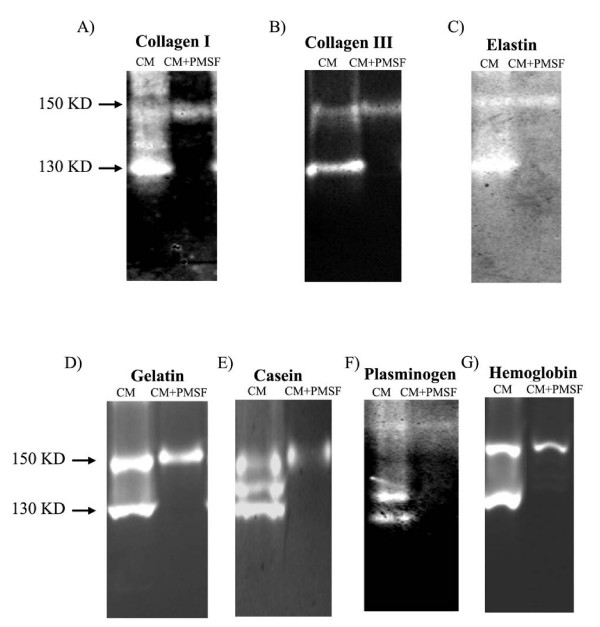
***Acanthamoeba *(T1) proteases degrade various protein substrates including extracellular matrix (ECM)**. Zymographic assays were performed using various substrates that constitute ECM or involved in ECM degradation. Note that proteases of AGE isolate degraded collagen I **(A)**, collagen III **(B)**, elastin **(C) **and plasminogen **(F)**. In addition, both proteases degraded gelatin **(D)**, casein **(E) **and haemoglobin **(G)**. CM is conditioned medium plus or minus the serine protease inhibitor, PMSF. Results are representative of three independent experiments.

The fibrillary collagens and elastin are structural proteins that are the major components of the ECM. To determine the ability of serine and metalloproteases to exhibit collagenase activities, zymographic assays were performed using collagen I and III as substrates. Both serine and metalloproteases degraded collagen I and III substrates (Fig. 5) confirming their collagenase activities. Additionally, both proteases degraded elastin (a major constituent of elastic fibers) in the zymographic assays, indicating that proteases from AGE isolate exhibit elastinolytic activities (Fig. 5). Among other substrates, these proteases exhibited degradation of casein and haemoglobin (Fig. 5).

### Both serine (130 KD) and metalloproteases (150 KD) of AGE isolate degrade plasminogen

Other modes of ECM degradation are tissue-type and urokinase-type plasminogen activators. The former pathway is mostly involved in fibrinolysis, while the latter is an important modulator in the pathophysiology of neuronal damage [8]. The activated urokinase converts pro-enzyme, plasminogen into plasmin, a serine protease involved in the ECM destruction by degrading fibrin. In this study, the zymographic assays revealed that both serine and metalloproteases from AGE isolate degraded plasminogen at neutral pH (Fig. 5).

### The metalloprotease activity can be separated from the serine protease activity by ion exchange chromatography

**Figure 6:**
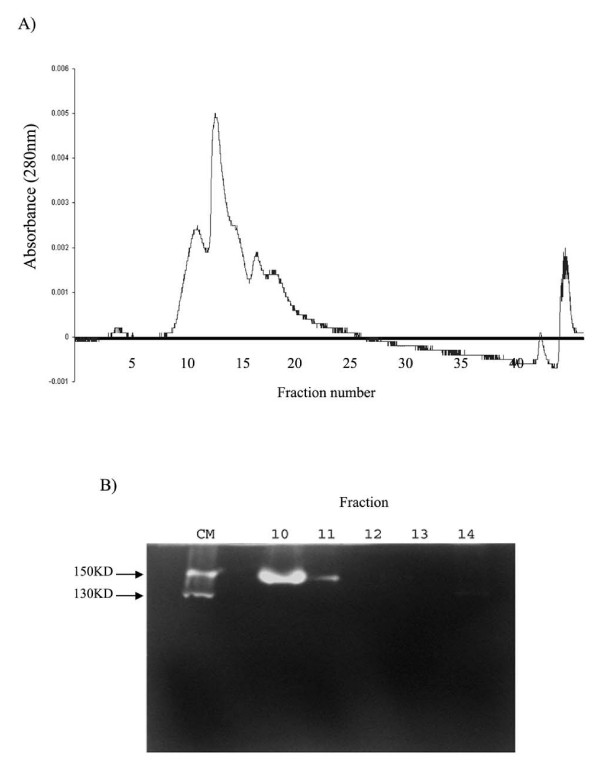
**Separation of metalloprotease by ion-exchange chromatography**. Proteins in CM from the AGE isolate were precipitated and fractionated using FPLC ion-exchange chromatography. **A) **A representative chromatogram showing a typical elution profile is shown. The collected fractions were tested for protease activity, and **B) **shows that fractions 10 and 11 (in the first major UV-absorbing peak) exhibited only metalloprotease activity (approx. Mw. 150 KD). Results are representative of three experiments.

In an attempt to obtain purified metalloprotease, FPLC ion-exchange chromatography was employed. The chromatograms in Fig. 6 show at least two major peaks and the corresponding column fractions exhibited protease activity. The eluted protein(s) in the first peak (fractions 10 and 11) contained metalloprotease activity (approx. Mw. 150 KD) (Fig. 6), separated apparently from the serine protease and sensitive to 1, 10-phenanthroline on gelatin gel zymography (data not shown). Of interest, the serine protease was observed in later fractions (data not shown).

## Discussion

*Acanthamoeba *granulomatous encephalitis is a serious CNS infection that almost always results in death. The pathophysiological complications involving the CNS most likely include invasion of the connective tissue, induction of extensive pro-inflammatory responses and neuronal damage leading to brain dysfunction. Thus it is reasonable to predict that *Acanthamoeba *proteases play important roles in AGE pathogenesis. Our previous studies have shown that *Acanthamoeba *proteases may play important roles in modulating blood-brain barrier permeability [9]. In this study, we further characterized *Acanthamoeba *proteases from an AGE isolate and developed methods to separate an extracellular metalloprotease from the extracellular serine protease.

The fact that a 150 KD extracellular metalloprotease is associated with an AGE isolate (and not keratitis isolate) suggests its possible involvement in the CNS pathology. To this end, we studied the properties of AGE isolate proteases on ECM degradation. In healthy brains, ECM comprises a major percent of the normal brain volume [10], which forms the basal lamina around the blood vessels. The ECM is constantly remodelled and provides critical structural and functional support to the neuronal tissue. These properties of ECM are tightly regulated by a family of mostly Ca^2+^-dependent Zn^2+^-containing endopeptidases (MMPs) [11,12]. The ECM plays important roles under normal physiological conditions in the development and maintenance of homeostasis in neuronal tissue. However in neurological disease states, ECM may undergo substantial modifications resulting in neuroinflammatory responses. Excessive ECM degradation affects neurovascular structural/functional properties that are highly destructive to the CNS functions. ECM is composed of both collagenous and non-collagenous glycoproteins and proteoglycans [11,12]. Here we showed that both the serine and metalloproteases of the AGE isolate exhibits collagen I and collagen III degradation suggesting that these proteases may facilitate amoebic migration into deeper tissues by degrading the ECM. These findings support previous findings which showed that *Acanthamoeba *exhibit collagenolytic activities [13]. Furthermore, both proteases exhibited elastinolytic activities. Elastin is the principal structural element that constitutes elastic fibres and is an important component of connective tissue. Previous findings have shown that elastase destroys ECM, which increases blood-brain barrier permeability resulting in the brain injury [14-16]. For example, injection of elastase into the cerebrospinal fluid (CSF) opened the blood-brain barrier in newborn piglets [17]. Our findings suggest that proteases of AGE isolate may play a similar role.

In addition to the aforementioned, the urokinase plasminogen activator system plays an important role in various neuronal diseases involving the CNS inflammation and/or pathology. For example, in bacterial meningitis, the uPA (urokinase-type plasminogen activator) or tPA (tissue-type plasminogen activator) are known to convert plasminogen, that is abundant in brain [18] into plasmin, which destroys ECM directly by degrading fibrin or by activating MMPs. We observed that both serine and metalloproteases directly degraded pro-enzyme, plasminogen suggesting that pathogenesis of AGE may involve the uPA/tPA system. Injections of plasmin results in increased capillary permeability [19]. At present, the precise role of the 150 KD metalloproteases remains unclear.

Our findings that CM-mediated HBMEC monolayer disruptions can be inhibited in the presence of PMSF suggest that the metalloprotease may not be responsible for HBMEC monolayer disruptions and may play a role in post-CNS invasion by amoebae. To this end, we have successfully developed methods for the separation of the metalloprotease from the serine protease of the AGE isolate, which should prove useful for subsequent purification and biochemical characterizations of this novel protease as well as to determine its role *in vivo*.

It is important to note that we only observed two proteases in our zymographic assays. However, the experimental conditions involved SDS detergent and there may be other proteases that are SDS-sensitive. Also, the identification of 130 KD serine protease is not novel. Previous studies have characterized a similar protease from an AK isolates [20-22] and again in the present study we observed 130 KD serine protease from an AK isolate belonging to T4 genotype. Thus it is reasonable to presume that the 130 KD serine protease is similar in both T1 and T4 isolates. Taken together, these studies have characterized the properties of a novel metalloprotease that only is produced by the AGE isolate. Future studies will precise determine its role in the virulence of AGE. Of interest, metalloproteases have been identified as important virulence factors in various opportunistic pathogens including *Vibrio vulnificus *[23], *Pseudomonas aeruginosa *[24], and *Aspergillus fumigatus *[25].

## Conclusion

In summary, these data show that both serine and metalloproteases secreted by AGE isolate (T1 genotype), exhibit properties to degrade ECM, which may facilitate amoebic invasion of the deeper lying tissues of the CNS. If future studies confirm that these proteases are indeed important in the pathology of AGE then they could be potential targets for the rationale development of therapeutic interventions.

## Methods

All chemicals were purchased from Sigma (Poole, Dorset, UK), unless otherwise stated.

### *Acanthamoeba *cultures

Two isolates of *Acanthamoeba *were used in the present study: 1) *A. castellanii *(ATCC 50494) belonging to T1 genotype was isolated from an AGE patient, and 2) *A. castellanii *(ATCC 50492)belonging to T4 genotype was isolated from a keratitis patient. For simplicity, the former is referred to as the AGE isolate and the latter is referred to as the AK isolate. Both isolates were grown in tissue culture flasks in PYG medium [proteose peptone 0.75% (w/v), yeast extract 0.75% (w/v) and glucose 1.5% (w/v)] at 30°C and the medium was refreshed 17–20 h prior to experiments as previously described [26]. This resulted in >95% *Acanthamoeba *in the trophozoite forms.

### Human brain microvascular endothelial cell cultures

The primary brain microvascular endothelial cells were isolated from human tissue and purified by fluorescent activated cell sorting (FACS). The purified cells were tested for endothelial characteristics, such as expression of endothelial markers, F-VIII, carbonic anhydrase IV and uptake of acetylated low density lipoprotein (AcLDL), indicating their endothelial origin and expression of gamma-glutamyl transpeptidase, indicating the brain origin (27). HBMEC were grown in RPMI-1640 containing 10% foetal bovine serum, 10% NuSerum, 2 mM glutamine, 1 mM pyruvate, penicillin (100 U/ml), streptomycin (100 U/ml), non-essential amino acids and vitamins at 37°C, 5% CO_2 _as previously described [27,28].

### Cytotoxicity assays

To examine the cytopathic potential of *Acanthamoeba *isolates, cytotoxicity assays were performed as previously described [26]. Briefly, HBMEC were grown to confluent monolayers in 24-well plates. *Acanthamoeba *(5 × 10^5 ^amoebae/well) were incubated with HBMEC monolayers in serum free medium (RPMI 1640 containing 2 mM glutamine, 1 mM pyruate and non-essential amino acids) in plates incubated at 37°C in 5% CO_2_. The HBMEC monolayers were observed periodically under a phase-contrast microscope for cytopathic effects for up to 24 h. After 24 h, the supernatants (termed conditioned medium, CM) were collected, and the monolayers were fixed and stained with hematoxylin to visualize HBMEC monolayer disruptions. In addition, supernatants were examined for HBMEC cytotoxicity by measuring lactate dehydrogenase (LDH) release (cytotoxicity detection kit; Roche Applied Science, Lewes, East Sussex, UK). Briefly, CM of co-cultures of *Acanthamoeba *and HBMEC were assessed for the presence of LDH, the release of which is considered as an estimate of cell death. The percentage LDH release was calculated as follows: [LDH activity in experimental sample (measured by optical density at 590 nm) - LDH activity in control samples/total LDH activity release - LDH activity in control samples × 100 = % cytotoxicity). Control samples were obtained from HBMEC or *Acanthamoeba *incubated alone. Total LDH activity release was determined by total HBMEC lysis with 1% Triton X-100 for 30 min at 37°C.

To determine the cytopathic effects of extracellular proteases released from intact *Acanthamoeba*, CM was produced by inoculating *Acanthamoeba *in serum free medium described above for 24 h. Next day, *Acanthamoeba *free supernatants, i.e., CM was collected by centrifugation and used for cytotoxicity assays as described above.

Some cytotoxicity assays were performed in the presence or absence of broad spectrum matrix metalloprotease (MMP) inhibitors, i.e., GM 6001 and GM 1489 (Merck Biosciences Ltd. Nottingham, England) or phenylmethanesulfonyl fluoride (PMSF), an irreversible inhibitor of serine proteases. Briefly, the protease inhibitors (PMSF, 2 mM final conc.; GM 6001 and GM 1489, 5 μM final conc.) were added to CM for 30 min and then mixtures were added to HBMEC monolayers.

### Zymography

Zymographic assays were performed to visualize and begin to characterize *Acanthamoeba *extracellular proteases as previously described [29]. Briefly, CM was mixed (1:1) with sample buffer (containing 4% sodium dodecyl sulfate (SDS) but without β-mercaptoethanol) and electrophoresed on SDS-polyacrylamide gels (SDS-PAGE) containing gelatin (1 mg/mL). After electrophoresis, gels were soaked in 2.5% Triton X-100 (w/v) for 60 min to remove the SDS. Finally, the gels were incubated in a developing buffer (50 mM Tris-HCl, pH 7.5, containing 10 mM CaCl_2_) at 37°C overnight, rinsed, and stained with Coomassie brilliant blue. Areas of gelatin digestion indicating protease activities are seen as non-staining regions in the gel. In some experiments, samples were pre-treated with PMSF (2 mM final concentration), or 1, 10-phenanthroline (10 mM final concentration) for 30 min. Because 1, 10-phenanthroline is a reversible inhibitor, it was also included in the developing buffer.

To determine the optimum temperature for *Acanthamoeba *protease activities, gels were incubated at 4°C, 10°C, 20°C, 30°C, 37°C, 42°C, 50°C and 65°C. For the determination of optimum pH, gels were incubated at pH ranging from 3 – 13. For pH 3 and 4, developing buffers were prepared using citrate-phosphate buffer (70 mM sodium citrate and 60 mM sodium phosphate) containing 10 mM CaCl_2_; for pH 5 and 6, developing buffers were prepared using N- [2-morpholino] ethanesulfonic acid (MES) containing 10 mM CaCl_2_; and for pH 7 – 13, developing buffers were prepared using 50 mM Tris containing 10 mM CaCl_2_.

In some experiments, we used extracellular matrix (ECM) proteins including collagen I (obtained from bovine achilles tendon; 1 mg/mL final conc.) dissolved in buffer A (50 nM TES {N-tris [hydroxymethyl]methyl-2-aminoethane-sulfonic acid}, pH 7.4 plus 0.36 mM CaCl_2_), collagen III (calf skin; 1 mg/mL final conc.) dissolved in buffer B (distilled H_2_O, pH 3 using acetic acid), elastin (bovine neck ligament; 1 mg/mL final conc.) dissolved in buffer C (200 mM Tris, pH 8.8) and plasminogen (rabbit plasma; 1 mg/mL final conc.) dissolved in buffer D (20 mM lysine, pH 7) as substrate for *Acanthamoeba *proteases. In addition we used casein (bovine milk; 1 mg/mL final conc.) dissolved in buffer E (60 mM Tris, 90 mM NaCl, pH7.5) and haemoglobin (bovine erthyrocytes; 1 mg/mL final conc.) dissolved in distilled H_2_O with gentle heat as substrate for *Acanthamoeba *proteases. All substrates were dissolved according to manufacturer's instructions (i.e., Sigma Labs).

### Ammonium sulphate precipitation

In an attempt to purify extracellular proteases of *Acanthamoeba*, (ca. 500 mL), CM was produced and proteins precipitated by adding ammonium sulphate (0.7 g/mL) slowly to the CM while stirring at 4°C to achieve 70% saturation. The mixture was stirred for an additional 25 min. The precipitate was recovered by centrifugation (10,000 × *g *for 10 min at 4°C) and dissolved in 500 μl of 0.02 M ammonium acetate buffer, pH 6.5. The concentrated proteins were dialysed against HPLC grade H_2_O overnight to remove residual ammonium sulphate.

### Ion-exchange chromatography

The dialysed precipitated proteins were applied to a DEAE-Sepharose fast flow beads column (16 cm length, 2 cm diameter) (Amersham Biosciences). The column was equiliberated with 100 mL of 0.02 M ammonium acetate buffer, pH 6.5 at a flow rate of 4 mL per min. Using a fast performance liquid chromatography system (Biorad, Hemel Hempstead, UK), proteins were eluted in 0.02 M ammonium acetate with a 0 to 1.0 M NaCl gradient. Proteins in the eluted fractions (6 mL) were freeze dried for long term storage. The lyophilised fractions were reconstituted in 1 mL of 50 mM Tris-HCl, pH 6.8 and tested for protease activities using zymographic assays.
